# Effects of *Lycium barbarum* polysaccharides supplemented to high soybean meal diet on immunity and hepatic health of spotted sea bass *Lateolabrax maculatus*

**DOI:** 10.3389/fimmu.2024.1333469

**Published:** 2024-02-06

**Authors:** Longhui Liu, Yanbo Zhao, Zhangfan Huang, Zhongying Long, Huihui Qin, Hao Lin, Sishun Zhou, Lumin Kong, Jianrong Ma, Zhongbao Li

**Affiliations:** ^1^ Fisheries College, Jimei University, Xiamen, China; ^2^ Fujian Provincial Key Laboratory of Marine Fishery Resources and Eco-environment, Xiamen, China

**Keywords:** *Lycium barbarum* polysaccharides, high soybean meal diet, spotted sea bass, Immunity, hepatic health

## Abstract

High soybean meal diet (HSBMD) decreased the immunity and damaged the liver health of spotted sea bass; in this study, *Lycium barbarum* polysaccharides (LBP) was added to HSBMD to explore its effects on the immunity and liver health. The diet with 44% fish meal content was designed as a blank control. On this basis, soybean meal was used to replace 50% fish meal as HSBMD, and LBP was added in HSBMD in gradient (1.0, 1.5, 2.0 g/kg) as the experimental diet. 225-tailed spotted sea bass with initial body weight of 44.52 ± 0.24 g were randomly divided into 5 groups and fed the corresponding diet for 52 days, respectively. The results show that: after ingestion of HSBMD, the immunity of spotted sea bass decreased slightly and hepatic tissue was severely damaged. And the addition of LBP significantly improved the immune capacity and protected the hepatic health. Specifically, the activities of serum lysozyme (LZM), immunoglobulin M (IgM), liver acid phosphatase (ACP) and alkaline phosphatase (AKP) were increased, and serum alanine aminotransferase (ALT) and aspartate aminotransferase (AST) activities were significantly decreased, and hepatic morphology was improved. In the analysis of transcriptome results, it was found that toll-like receptor 3 (TLR3) and toll-like receptor 5 (TLR5) were down-regulated in toll-like receptor signaling pathway. And LBP may protect hepatic health by regulating Glycolysis/Gluconeogenesis, Insulin signaling pathway, Steroid biosynthesis and other glucolipid-related pathways. In conclusion, the addition of LBP in HSBMD can improve the immunity and protect the hepatic health of spotted sea bass, and its mechanism may be related to glucose and lipid metabolism.

## 1 Introduction

The continuous growth of aquaculture production to meet the growing demand for seafood in the world is necessary (1). Over the past three decades, aquaculture has produced about half of the aquatic products consumed by humans, and fish have provided at least 15% of the per capita animal protein intake for more than 4.5 billion people (2, 3). Aquaculture is particularly dependent on fishmeal, especially carnivorous Marine fish farming (4). Unfortunately, aquaculture that relies on fishmeal as its main protein source is not sustainable due to high demand and unstable sources (5). At present, soybean meal, a renewable plant protein with high protein content and relatively balanced amino acid composition, is regarded as the most promising protein source to replace fish meal (6). However, many studies have reported various disadvantages of a diet with varying amounts of soybean meal for fish (7–9). For example, high soybean meal diet (HSBMD) can cause a series of problems in turbot (*Scophthalmus maximus*), including slower growth, tissue degeneration, inflammation and other physiological and metabolic disorders (10). It is worth mentioning that the above symptoms are particularly obvious in carnivorous fish (11, 12).

*Lycium barbarum* is a plant in the Solanaceae family (*Solanaceae Juss*) that is widely distributed in northwestern China; and it was also introduced in North and South America and Western Europe (13). *Lycium barbarum* is a common tonic and traditional Chinese medicine in China, because it is rich in nutrients and active substances, including polysaccharides, polyphenols, amino acids, trace elements and vitamins (14). In past studies, *Lycium barbarum* polysaccharides (LBP) had been identified as one of the active ingredients responsible for the biological activities (15). Many pharmacological and phytochemical studies have shown that LBP has antioxidant (16), immunomodulatory (17), hypoglycemic (18), anti-inflammatory (19) and liver protection (20). Meanwhile, in our previous study, LBP was found to have excellent effects in regulating hepatic lipid metabolism (21). Thus, LBP may be able to mitigate the adverse effects of HSBMD on the body through the aforementioned effects.

Spotted sea bass (*Lateolabrax maculatus*) is a widely farmed economic fish which is loved by people because of its delicious meat and rich nutrition. According to statistics, the yield of spotted sea bass in 2022 was 21.81 ten thousand tons, and the number of breeding was huge (22). Spotted sea bass is a typical carnivorous fish. As a matter of course, HSBMD causes it to produce a series of related discomfort reactions (23). Therefore, this experiment explored the effects of LBP supplemented to HSBMD on immunity and hepatic health of spotted sea bass. It also provides a theoretical basis for LBP as a feed additive to protect the hepatic health of aquaculture animals.

## 2 Materials and methods

### 2.1 LBP preparation and characterization

LBP was purchased from Xi’an Shengqing Biotechnology Co., LTD, China. The total sugar content is 80.90% (phenol-sulfuric acid method). The sample were degreased and decolorized refer to the methods of Cui (24) and Wu (25). Two compositions were isolated from LBP. The molecular weight and yield of composition 1 were 11.343 kDa and 31.4%, respectively, and including galactose (0.08%), mannuronic acid (5.19%), arabinose (0.21%), guluronic acid (7.04%), galacturonic acid (16.57%) glucuronic acid (9.36%) and glucose (61.55%). The molecular weight and yield of composition 2 were 35.508 kDa and 20.9%, respectively, and including glucose (96.59%), galactose (1.01%), arabinose (1.69%), mannose (0.44%), and xylose (0.27%). More detailed results on the preparation and characterization of LBP can be found in previous report (21).

### 2.2 Experimental diets

Five dietary formulations with crude protein about 45% and crude fat about 12% were designed according to the nutritional requirements of spotted sea bass (26). 44% fish meal (0% soybean meal) was taken as healthy control and labeled as LS group. In addition, half of the fishmeal was replaced with 40% soybean meal and labeled as HS group; accordingly, on the basis of HS group, LBP was added (1.0, 1.5, 2.0 g/kg) and labeled as HL1, HL2, HL3, respectively. Flour was used for balancing among the groups (**Table 1**).

**Table 1:** Nutritional composition of experimental diet (Dry matter basis).

Ingredients	Group/Contents (g/kg)				
	LS	HS	HL1	HL2	HL3
**Fish meal**	440	220	220	220	220
**Soybean meal**	0	400	400	400	400
**Casein**	110	110	110	110	110
**Flour**	345	149	148	147.5	147
**Fish oil**	35	50	50	50	50
**Soybean oil**	25	25	25	25	25
Mineral premix^a^	6	6	6	6	6
**Antioxidant**	3	3	3	3	3
**Ca(H_2_PO_4_)_2_**	12	12	12	12	12
Vitamin premix^b^	8	8	8	8	8
**Choline**	6	6	6	6	6
**Methionine**	0	1.0	1.0	1.0	1.0
**Lecithin**	10	10	10	10	10
**LBP**	0	0	1.0	1.5	2.0
**Total**	1000				

The proportion of nutrients of the main ingredients in the feed: Soybean meal: Crude fat, 1.9%, Crude protein, 44.2%; Flour: Crude fat, 3%, Crude protein, 13%; Fish meal: Crude fat, 8.4%, Crude protein, 67%.

a Mineral premix: MnSO_4_·4H_2_O 50 mg/kg, MgSO_4_·H_2_O 4000 mg/kg, KI 100 mg/kg, CoCl_2_(1%) 100 mg/kg, CuSO_4_·5H_2_O 20 mg/kg, FeSO_4_·H_2_O 260 mg/kg, ZnSO_4_·H_2_O 150 mg/kg, Na_2_SeO_3_(1%) 50 mg/kg.

b Vitamin premix: thiamine 25 mg/kg, riboflavin 45 mg/kg, pyridoxine hydrochloride 20 mg/kg, Vitamin B12 0.1 mg/kg, Vitamin K3 10 mg/kg, inositol 800 mg/kg, pantothenic acid 60 mg/kg, nicotinic acid 200 mg/kg, folic acid 20 mg/kg, biotin 1.2 mg/kg, vitamin A acetate 32 mg/kg, Vitamin D3 5 mg/kg, α-tocopherol 120 mg/kg, ethoxyquin 150 mg/kg.

The raw feed materials were thoroughly crushed in the crusher. According to the principle of mixing from small to large, the various ingredients in the formula were fully mixed, and then the fish oil and soybean oil were mixed, and finally the appropriate amount of water (about 35% of the weight of the feed) was added, and the particles were squeezed into 5 mm particle size through the granulator. In particular, LBP was completely soluble in water when added. The prepared feed was dried to a moisture content of 10% in a constant temperature oven at 55°, subsequently cooled to room temperature, and finally stored at -20° for future utilization.

### 2.3 Experimental fish and feeding management

This experiment was approved by the Animal Ethics Committee of Jimei University (Grant No. JMU202103009). The fish were purchased from commercial fisheries in Fujian, China. The culture experiment was carried out in the seawater test field of Jimei University, Xiamen, China. And the experiment period was 52 days. Before the start of the study, the experiment site, temporary fish tank (1700 L) and experimental fish tank (160 L) were washed, disinfected and aerated, and the fish were temporarily raised for two weeks to adapt to the environment. During the temporary rearing, fish were fed to apparent satiety twice a day at 8:30 and 17:30. In addition, about 35% of the water was changed half an hour after the evening feeding was completed.

At the end of the temporary culture, the experimental fish was anesthetized with 150 mg/L eugenol after fasting for 24 hours (27). A total of 225 fish with similar size (44.52 ± 0.24 g) were randomly assigned to 15 fish tanks and divided into 5 groups with three replicates per group.

Feeding management during the breeding experiment was roughly the same as that of temporary rearing, and each group was fed the corresponding labeled feed separately. All tanks were connected, the temperature of the water controlled by the hot cycle heater was 27.0 ± 0.2°C, the dissolved oxygen was maintained at 7 mg/L, and the PH was maintained between 7.8 and 8.2.

### 2.4 Sample collection

Similarly, the experimental fish was fasted for 24 hours and anesthetized with 150 mg/L eugenol. Eleven fish were randomly selected from each tank, serum samples were collected by tail vein sampling method, and the serum was separated by centrifugation (836×g, 10min, 4°C) after 16 hours at 4°C, and stored at -80°C for later use. After that, the fish were humanely killed, the livers were collected, and 9 liver samples were briefly stored in liquid nitrogen, and then stored at -80°C. Among them, 5 liver samples were used for the detection of related enzyme activities, and 4 liver samples were used for transcriptome analysis. The remaining 2 samples were fixed with 4% paraformaldehyde for histological observation.

### 2.5 Analysis of immune parameters

In this study, the immune-related indicators included serum lysozyme (LZM), serum acid phosphatase (ACP), alkaline phosphatase (AKP), immunoglobulin M (IgM), and hepatic ACP and AKP. The principle of LZM activity detection is that LZM exhibited the ability to enzymatically degrade peptidoglycan present on the bacterial cell wall, resulting in bacterial lysis. This concentration gradually decreased while light transmittance increased (28). ACP and AKP can decompose phenylene disodium phosphate to produce free phenol and phosphoric acid, phenol reacts with 4-amino-antipyrine in alkaline solution to oxidize red quinone derivatives. So, the enzyme activities were determined by colorimetry (29). The principle of IgM activity detection is that competition method. In short, samples were added to the enzyme-labeled pore pre-coated with antibodies, and biotin-labeled recognition antigen was added. The two compete with solid phase antibodies to form an immune complex. After the unbound biotin antigen was washed away, avidin-HRP was added to bind to the biotin antigen, and color changes were generated under certain conditions. The light absorption values are negatively correlated with the antigen concentration (30). All kits were provided by Nanjing Jiancheng Biotechnology Co., LTD. (Item No: LZM: A050-1-1, ACP: A060, AKP: A059-2).

### 2.6 Analysis of related parameters of hepatic injury

In this study, hepatic injury related indexes included serum Alanine aminotransferase (ALT), Aspartate aminotransferase (AST) and liver ALT, AST, as well as observation and analysis of hepatic morphology. Both ALT and AST activities were operated as instructed, color reactions occurred, absorbances were measured at specific wavelengths, and enzyme activity was calculated (31). (Item No: ALT: C009-2-1, AST:C010-2-1).

#### 2.6.1 Hepatic morphological analysis

The hepatic morphology was observed by hematoxylin-eosin staining. In short, samples were fixed in 4% paraformaldehyde for 24 h after collection, fully rinsed with 75% ethanol solution, and sequentially soaked in 70%, 95% and 100% concentration of ethanol solution for dehydration. After dehydration, samples were transferred to 1:1 xylene ethanol solution for 20 minutes and xylene paraffin mixture for 30 minutes. Finally, the samples were quickly embedded at 60°C. After the samples were cooled and solidified, they were sliced by a microtome (RM2016, LEICA, Germany), spread in warm water, collected by slides and dried in a constant temperature oven at 55°C for 8h. The dried sections were deparaffinized in xylene and ethanol solutions and covered with coverslips after staining with hematoxylin and eosin solutions, respectively. A forward white light microscope (Eclipse Ci-L, Nikon, Japan) was used to photograph and analyze these slices. And these slices were observed in various multiples (200×) with CaseViewer Ver 2.2 (The Digital Pathology Corp., Hungary).

Fifteen liver section samples (three replicates per group) were randomly selected for quantitative analysis of fat vacuoles. Fiji (ImageJ-win64) was used for image analysis and processing. In short, after region-of-interest (ROI) was selected on the original image, a manual threshold was further selected to mark the fat vacuoles in the samples, calculate and output the IntDen value, and use the IntDen value of the LS group samples as the control, the relative area of fat vacuoles in each group was calculated. The obtained results were processed and mapped according to Method in 2.8 (32, 33).

### 2.7 Hepatic transcriptome analysis and RT-qPCR

#### 2.7.1 Total RNA extraction and detection

Total RNA was extracted by TRIzol kit, RNA purity and concentration were detected by NanoDrop 2000 spectrophotometer (manufacturer Thermerfly), and RNA integrity was detected by Agient2100 or LabChip GX.

#### 2.7.2 Library construction

Once the samples were qualified, a sequencing library was generated using NEBNext^®^Ultra™ RNA library preparation using Illumina^®^ (NEB USA) and an index code was added to the attribute sequence of each sample. In short, eukaryotic mRNA was enriched with magnetic beads with Oligo (dT), and mRNA was randomly interrupted by Fragmentation Buffer. The first cDNA strand and the second mRNA strand were synthesized using mrna as template, and cDNA purification was performed. After purification, the double-stranded cDNA was end-repaired, A-tail was added, and sequencing connectors were connected. Then AMPure XP beads (Beckman Coulter, Beverly, USA) were used for fragment size selection, and the cDNA library was enriched by PCR.

#### 2.7.3 Library quality control

After the library was constructed, the Qubit 3.0 fluorometric quantifier was used for preliminary quantification, and the concentration of c should exceed 1 ng/ul. Then the Qsep400 high-throughput analysis system was used to detect the inserted fragments of the library. After meeting the expectation, the effective concentration of the library (effective concentration > 2 nM) was accurately quantified by q-PCR method. To ensure the quality of the library.

#### 2.7.4 Sequencing

The Illumina NovaSeq6000 sequencing platform was used for PE150 mode sequencing.

#### 2.7.5 Transcriptome assembly and gene functional annotation

Tanscriptome assembly was accomplished based on the left.fq and right.fq using Trinity (min_kmer_cov set to 2 by default and all other parameters set default) (34). Gene function annotation was based on the following database: NR (NCBI non-redundant protein sequences), Pfam (Protein family), KOG/COG (Clusters of Orthologous Groups of proteins), Swiss-Prot (A manually annotated and reviewed protein sequence database), KEGG (Kyoto Encyclopedia of Genes and Genomes), GO (Gene Ontology).

#### 2.7.6 Quantification of gene expression levels

The level of gene expression in each sample was estimated by RSEM (35).

#### 2.7.7 Differential expression analysis

Based on the Count value of genes in each sample, DESeq2 (36) software was used to screen differential gene expression, and Fold Change ≥ 2 and FDR < 0.01 were used as screening criteria. All of the above analysis was performed passing BMKCloud (www.bioloud.net).

#### 2.7.8 RT-qPCR

RT-qPCR reaction was measured by SYBR Green I chimeric fluorescence method. Kits were purchased from Nanjing Vazyme Biotech Co., Ltd., China (Q711-02/03). Using β-actin as internal parameter, and, according to the results of transcriptomic analysis, 5 genes were randomly selected from the common differential genes of the three groups (LS, HS, HL1) for RT-qPCR reaction. Primer information was recorded in **Table 2**. Specifically, 2 μl template cDNA, 10 μL of 2×ChamQ Universal SYBR qPCR Master Mix were added to the reaction system. 0.4 μL of forward and reverse primer (10 μM), 7.2 μL RNase-free ddH_2_O. After preparation, the system was amplified by fluorescent quantitative PCR (LC480, Roche, Switzerland). The reaction conditions were: predenaturation at 95°C for 30 seconds, then reaction at 95°C for 10 s, 60°C for 30 s for 40 cycles; finally, the dissolution curves were collected under the reaction conditions of 95°C 15 s, 60°C 60 s and 95°C 15 s respectively. The relative gene expression between groups was calculated by 2-ΔΔCt.

**Table 2:** Information of primers.

Gene	Primer sequence (5’-3’)	Annealing temperature (°)
β-actin	F:AACTGGGATGACATGGAGAAG R:TTGGCTTTGGGGTTCAGG	60
TRINITY_DN1011_c0_g1	F:ACATCGCATCACTTCACTGC R:AGGACTTGGAACTGAGGTGG	60
TRINITY_DN1273_c0_g1	F:CTGACAGCCGGGACACTTT R:GCACTGTGACCCCTTTCATC	60
TRINITY_DN13763_c0_g1	F:CCAGTTCAGCGTGTATCAGC R:GGCTGGGGAGAGATCAAACT	60
TRINITY_DN14696_c0_g1	F:ATGGCTTTCTCCCTGCTTCT R:GCAAAGACAAGCACAGTGGA	60
TRINITY_DN17812_c0_g4	F: GGCGCATGGTGTTCAAGTAA R: GGGGCGAACTCAACTTTACC	60

F means forward primer, while R means reverse primer.

### 2.8 Data statistics and analysis

Average values of experimental data were calculated, expressed as mean ± SD. The difference between the two control groups (HS and LS) was analyzed by independent sample T test. The LBP intake group (HL1, HL2 and HL3) was compared with the two control groups, and one-way analysis of variance was used. All statistical analysis were performed using SPSS Ver26 software. When *P* < 0.05, the difference was significant and Duncan multiple range test was performed. All figures were produced by GraphPad Prism 8 except transcriptomic analysis.

## 3 Results

### 3.1 Analysis of immune parameters

**Figure 1:**
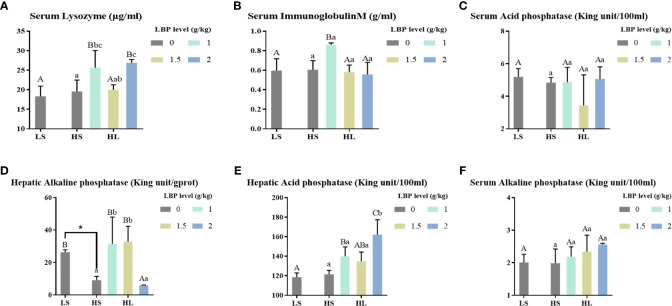
Results of immune-related enzyme activity index. Letters in corner marks **(A–F)** represent activity of serum lysozyme (serum LYZ, **A**), serum immunoglobulin M (serum IgM, **B**), serum acid phosphatase (serum ACP, **C**), hepatic alkaline phosphatase (hepatic AKP, **D**), hepatic acid phosphatase (hepatic ACP, **E**), serum alkaline phosphatase (serum AKP, **F**), respectively. The discrepancy between the LS group and HS group is indicated by * (P<0.05). Capital letters represent the discrepancy between LBP intake groups and LS group, while different letters indicate significant variation (P<0.05). Lowercase letters represent the discrepancy between LBP intake groups and HS group, while different letters indicate significant variation, too (P<0.05). Same to the following figures.

The results of immune-related indexes are shown in **Figure 1**. Compared with the LS group, the high soybean meal diet significantly decreased the hepatic AKP activity (*P* < 0.05). However, after adding LBP, the activities of serum LZM and IgM, hepatic AKP and ACP were significantly increased (*P* < 0.05). Among them, when the supplemental level was 1 g/kg, the serum LZM activity was significantly increased compared with the two control groups (*P* < 0.05), the serum IgM and hepatic ACP activities were significantly increased compared with the LS group (*P* < 0.05), and the hepatic AKP activity was significantly higher than the HS group (*P* < 0.05). In addition, when the supplemental level was 2 g/kg, the hepatic ACP activity was significantly higher than all groups (*P* < 0.05).

### 3.2 Analysis of related parameters of hepatic injury

#### 3.2.1 Enzyme activity index

**Figure 2:**
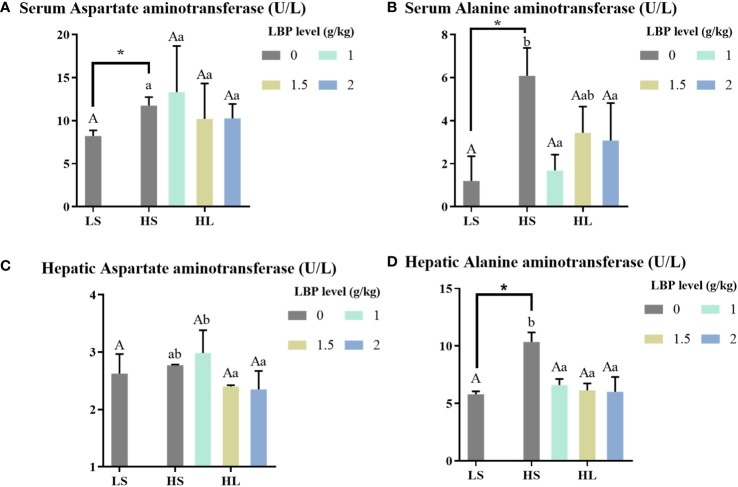
Results of hepatic injury-related enzyme activity indexes. Letters in corner marks **(A–D)** represent activity of serum aspartate aminotransferase (serum AST, **A**), serum alanine aminotransferase (serum ALT, **B**), hepatic aspartate aminotransferase (hepatic AST, **C**), hepatic alanine aminotransferase (hepatic ALT, **D**), respectively. The discrepancy between the LS group and HS group is indicated by *(P<0.05). Capital letters represent the discrepancy between LBP intake groups and LS group, while different letters indicate significant variation (P<0.05). Lowercase letters represent the discrepancy between LBP intake groups and HS group, while different letters indicate significant variation, too (P<0.05).

The results of liver injury-related enzyme activity indexes are shown in **Figure 2**. Compared with the whole fish meal diet, the intake of HSBMD caused hepatic damage, which was reflected in serum AST and ALT, and hepatic ALT activity was significantly increased (*P* < 0.05). After the addition of LBP, there was no significant difference between serum AST and the two control groups, but serum ALT and hepatic ALT were significantly decreased compared with HS group (*P* < 0.05), and even recovered to the level close to that in LS group. Among them, hepatic ALT activity of all LBP supplemental groups was significantly lower than HS group (*P* < 0.05), and had no significant difference compared with LS group. When LBP supplemental levels were 1 and 2 g/kg, the serum ALT activity was significantly lower than HS group (*P* < 0.05), but no significant difference was found between LS group and HS group.

#### 3.2.2 Hepatic morphological analysis

**Figure 3:**
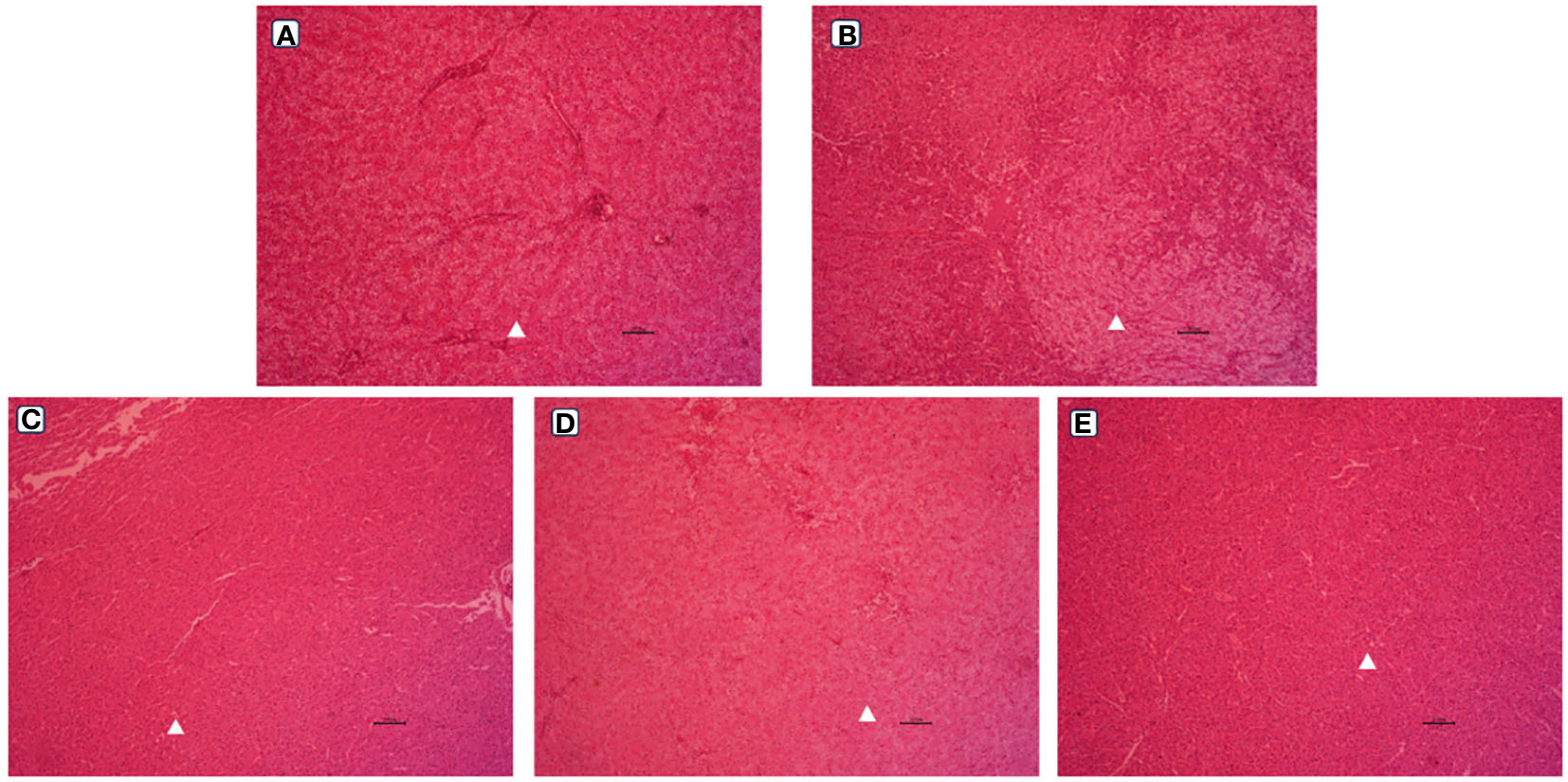
Hepatic morphological (100 ×) of spotted sea bass. Letters in corner marks A to E represent LS group **(A)**, HS group **(B)**, HL1 group **(C)**, HL2 group **(D)**, and HL3 group **(E)**, respectively. The triangular marks indicate balloon-like deformation and fat vacuoles.

**Figure 4:**
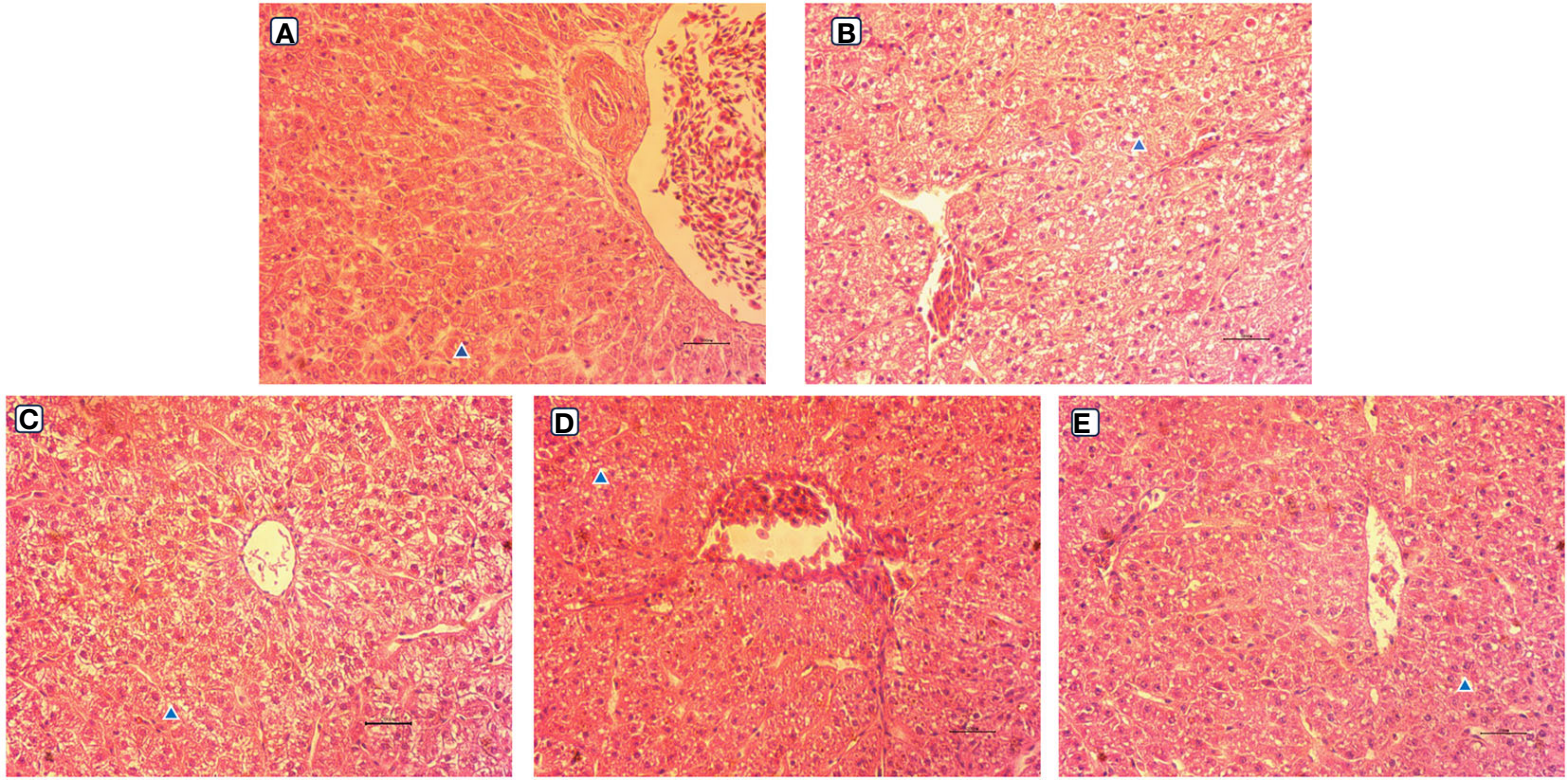
Hepatic morphological (400 ×) of spotted sea bass. Letters in corner marks A to E represent LS group **(A)**, HS group **(B)**, HL1 group **(C)**, HL2 group **(D)**, and HL3 group **(E)**, respectively. The triangular marks indicate balloon-like deformation and fat vacuoles.

The observation results of the hepatic morphology are shown in **Figure 3**. Few fat vacuoles and balloon deformation were observed in the LS group, and overall liver morphology was normal (**Figures 3A**, **4A**). However, after ingestion of HSBMD, a large number of vacuoles and balloon deformation occurred in the hepatic, and the cytoplasm was subsequently loosened, showing a very unhealthy state overall (**Figures 3B**, **4B**). LBP supplementation could improve the hepatic damage caused by HSBMD to a certain extent. Compared with the HS group, fewer lesions were observed in the LBP addition group, and fewer fat vacuoles and balloon deformation were observed. When the LBP supplemental level was 1g/kg, the hepatic health of the HL1 group was close to LS group (**Figures 3C**, **4C**). However, when LBP adding amount of 1.5 and 2.0 g/kg, fat vacuoles and balloon deformation increased slightly (**Figures 3D, E**, **4D, E**).

**Figure 5:**
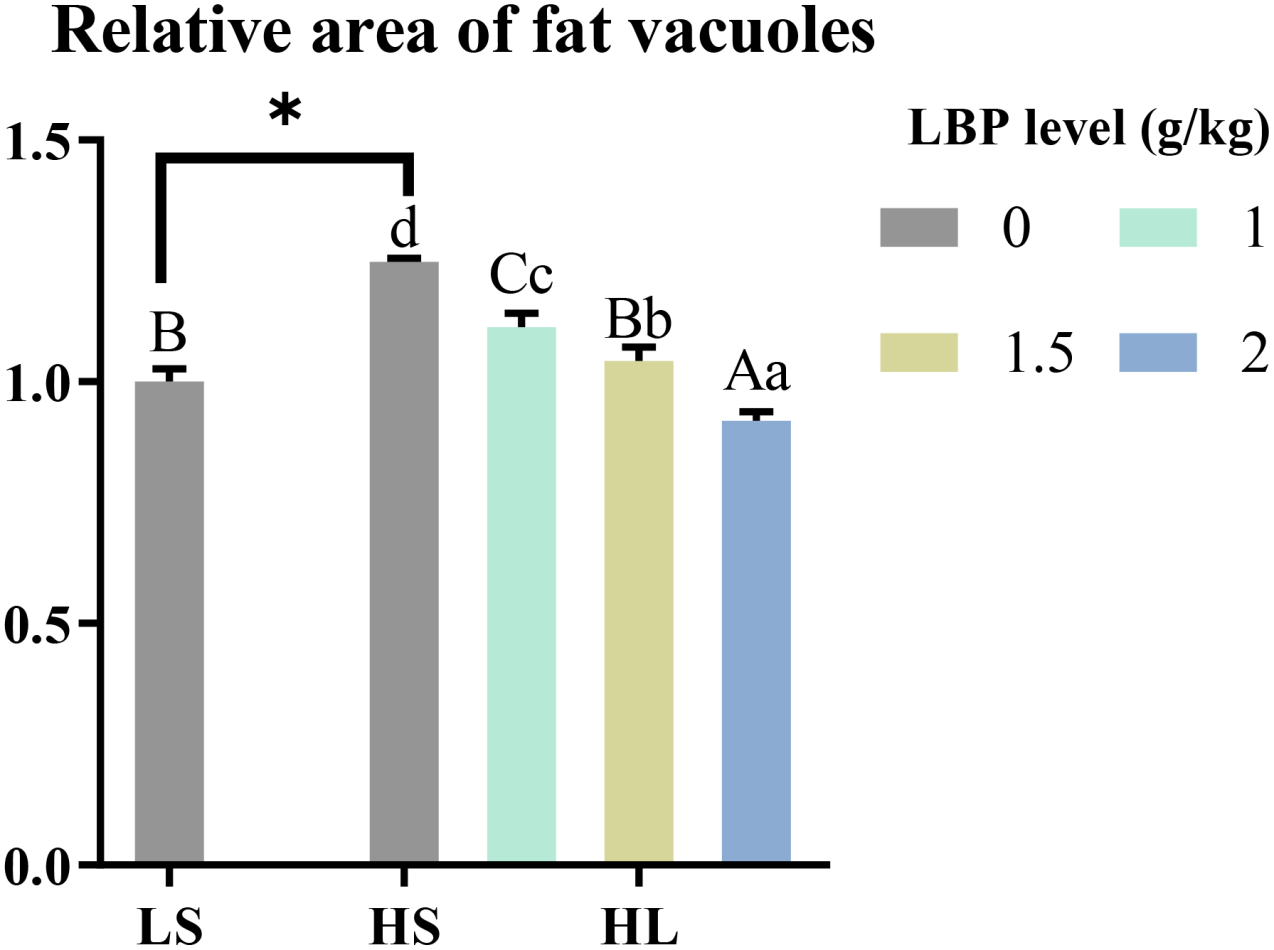
Results of the relative area of fat vacuoles. The discrepancy between the LS group and HS group is indicated by *(P<0.05). Capital letters represent the discrepancy between LBP intake groups and LS group, while different letters indicate significant variation (P<0.05). Lowercase letters represent the discrepancy between LBP intake groups and HS group, while different letters indicate significant variation, too (P<0.05).

The results of the relative area of fat vacuoles between the groups showed in **Figure 5**. Compared with LS group, the relative area of fat vacuoles in HS group was significantly increased (*P* < 0.05). After adding LBP, the relative area of fat vacuoles was decreased significantly (*P* < 0.05). There was a tendency to decrease with the increase of LBP addition.

### 3.3 Summary of results of liver transcriptome analysis

According to the above analysis results, we selected LS, HS, and HL1 groups for transcriptome sequencing, and compared the two groups. A total of three comparison groups were: LS vs HS, HS vs HL1 and LS vs HL1. A total of 57.43 Gb of Clean Data were obtained, and the Clean Data of all samples reached 5.86 Gb, and the percentage of Q30 base was 94.35% or above. A total of 44,470 Unigenes were obtained after assembly. There are 15,484 Unigenes pieces with a length of more than 1 kb. After functional annotation of Unigenes, 21,753 Unigenes annotation results were obtained.

#### 3.3.1 Differentially expressed genes

**Figure 6:**
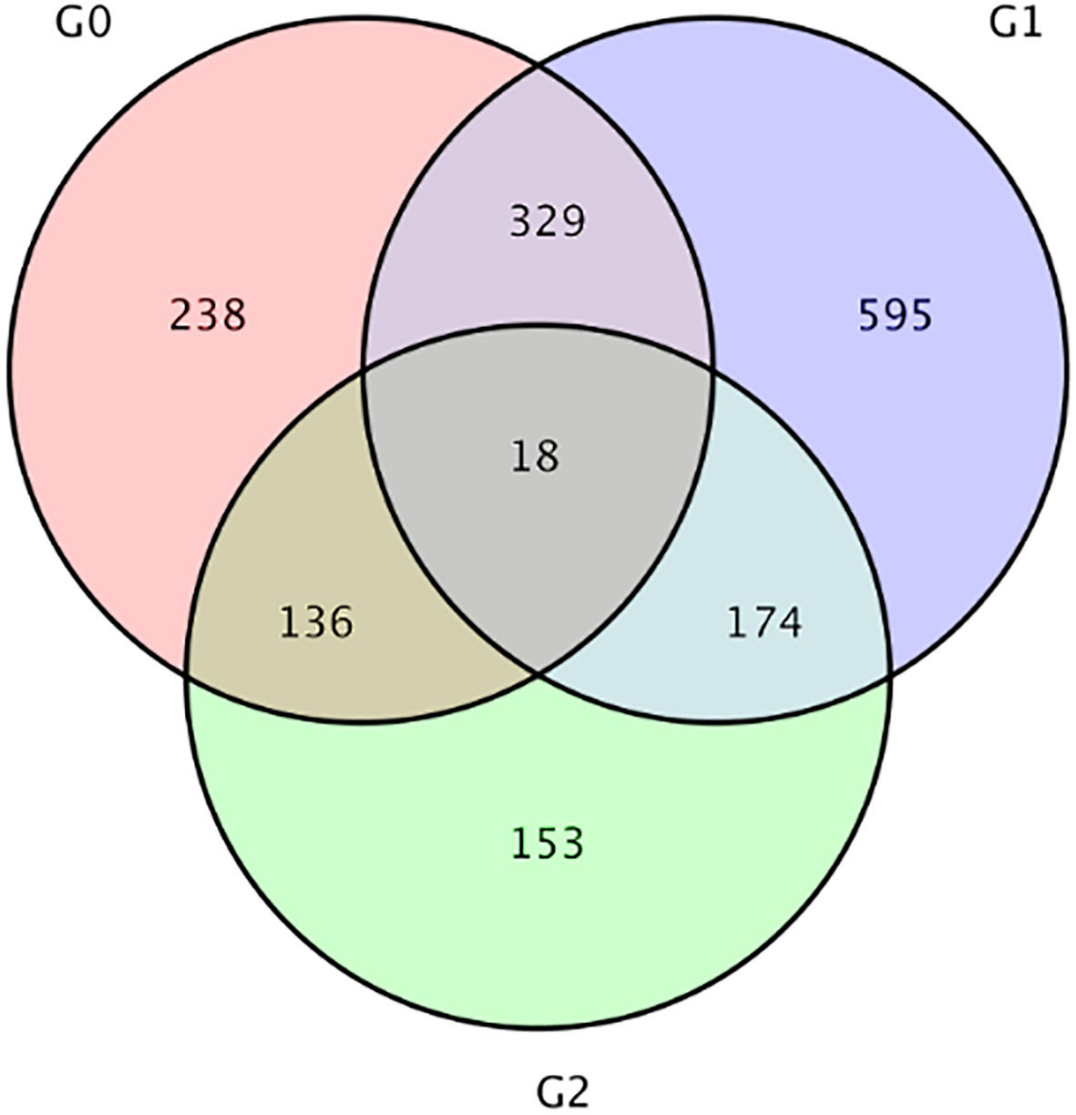
Venn diagram of differential genes. G0: HS vs HL1, G1:LS vs HL1, G2: LS vs HS. The numbers on each region represent the number of genes in the corresponding classification, where overlapping regions represent the number of differential genes shared between related combinations in the reg.

Compared with LS group, a total of 481 DEGs were observed in HS group, including 230 up-regulated and 251 down-regulated, and 1116 DEGs were observed in HL1 group, including 540 up-regulated and 576 down-regulated. Compared with the HS group, 721 DEGs were identified in the HL1 group supplemented with LBP, of which 419 were up-regulated and 302 were down-regulated (**Table 3**). In addition, there were a total of 18 DEGs between the three comparison groups. The specific number of total DEGs between the two comparison groups are shown in **Figure 6**.

**Table 3:** DEGs of between groups.

Group	All DEGs	Up-regulated	Down-regulated
LS vs HS	481	230	251
HS vs HL1	721	419	302
LS vs HL1	1116	540	576

#### 3.3.2 GO classification enrichment analysis

**Figure 7:**
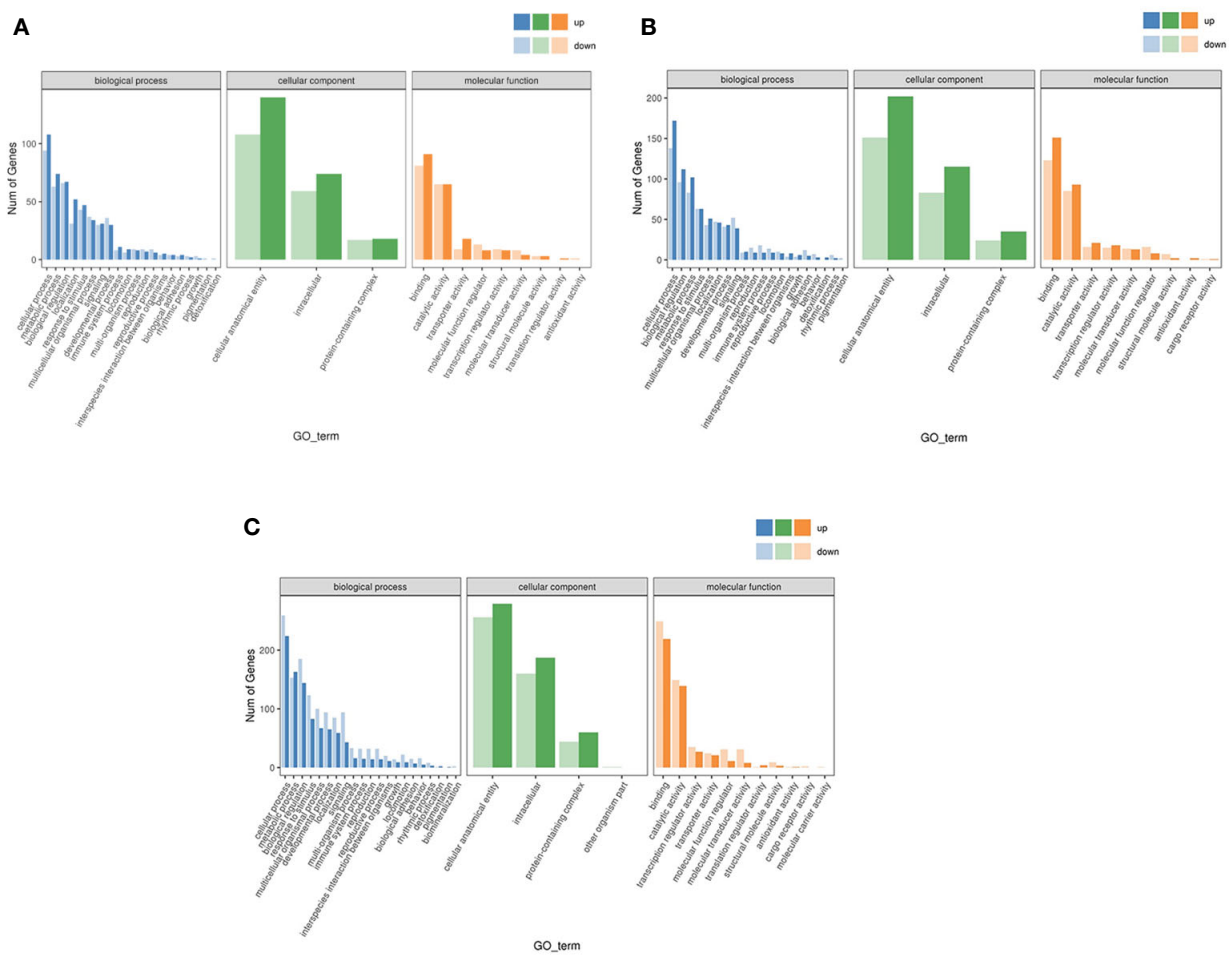
Differential expression gene GO annotation classification statistical map. Letters in corner marks **(A–C)** represent LS vs HS **(A)**, HS vs HL1 **(B)**, LS vs HL1 **(C)**, respectively. The horizontal coordinate is the GO classification, the vertical coordinate is the number of genes, and the different colors represent the different primary classification.

In the three branches of biological process, cellular component and molecular function, the number of up-regulated genes in LS vs HS comparison group were more than down-regulated genes. And biological process was concentrated in terms of cellular process, metabolic process, and localization. Cellular component was focused on cellular anatomical entity and intracellular, and molecular functions was focused on binding and transporter activities. Similarly, HS vs HL1 comparison group showed similar results to LS vs HS in terms of biological process, cellular component, molecular function and their secondary classification level. However, when comparing the HL1 group with the LS group, different results emerged. In the biological process and cellular component of HL1 group, the number of up-regulated genes were greater than the number of down-regulated genes, and they were concentrated in the above levels. But, it was interesting to note that the up-regulated number of binding and transporter activities in molecular function were smaller than the down-regulated number (**Figure 7**).

**Figure 8:**
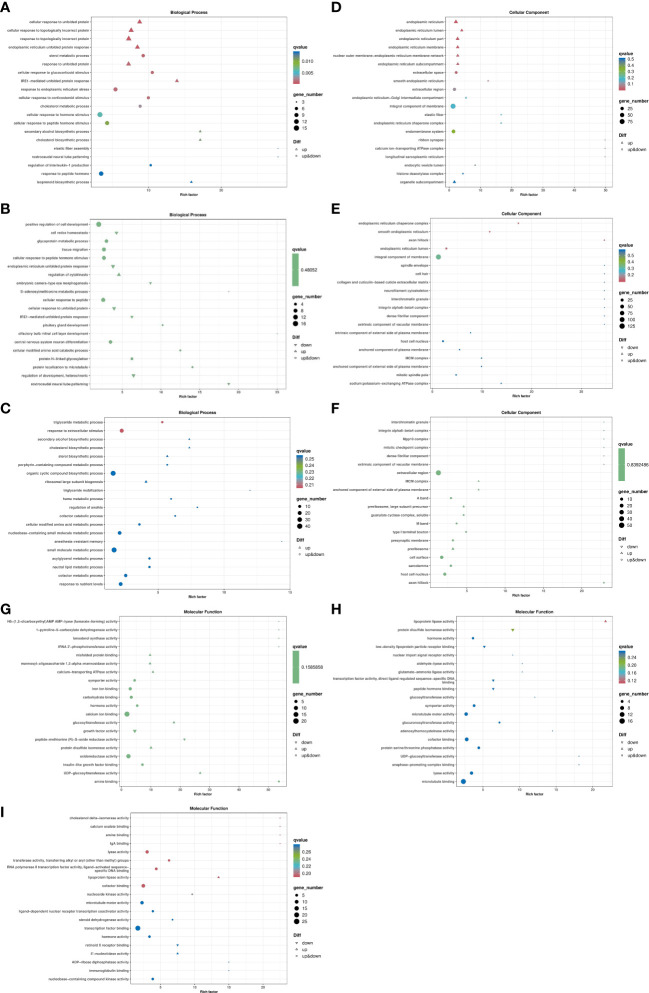
Bubble map of differentially expressed gene GO enrichment. Letters in corner marks **(A–I)** represent LS vs HS biological process **(A)**, HS vs HL1 biological process **(B)**, LS vs HL1 biological process **(C)**, LS vs HS cellular component **(D)**, HS vs HL1 cellular component **(E)**, LS vs HL1 cellular component **(F)**, LS vs HS molecular function **(G)**, HS vs HL1 molecular function **(H)**, LS vs HL1 molecular function **(I)**, respectively. The horizontal coordinate is Rich factor, which represents the ratio of the proportion of genes annotated to a pathway in differential genes to the proportion of genes annotated to that pathway in all genes, and the vertical coordinate represents the GO annotation term. The higher the enrichment factor, the more significant the enrichment level of differentially expressed genes in the GO term. Upper triangle, lower triangle and circle respectively represent the differential expression of genes enriched into the pathway, in which the upper triangle indicates that all genes enriched into the pathway are up-regulated genes, the lower triangle indicates that all genes enriched into the pathway are down-regulated genes, and the circle indicates that genes enriched into the pathway are both up-regulated and down-regulated genes. The color of the shape represents qvalue, the smaller the qvalue, the lower the qvalue. It indicates that the enrichment of differentially expressed genes in this GO term is more reliable. The size of the shape indicates the number of genes enriched in the GO term, and the larger the shape, the more genes.

ClusterProfiler was used to conduct enrichment analysis of biological process, cellular component and molecular function by hypergeometric testing method. The results showed that: in the biological process, LS vs HS comparison group differentially enriched in cellular response to unfolded protein, response to topologically incorrect protein and response to unfolded protein and other terms, and the differential genes showed up-regulation in these terms. The term difference in LS vs HL1 comparison group mainly included the Triglyceride metabolic process and response to extracellular stimulus. In the HS vs HL1 comparison group, cell redox homeostasis, endoplasmic reticulum unfolded protein response and cellular response to unfolded protein terms were down-regulated. In particular, cellular response to unfolded protein was up-regulated in the LS vs HS comparison group. The specific enrichment term of differentially expressed genes are shown in **Figure 8**.

#### 3.3.3 KEGG annotation analysis

**Figure 9:**
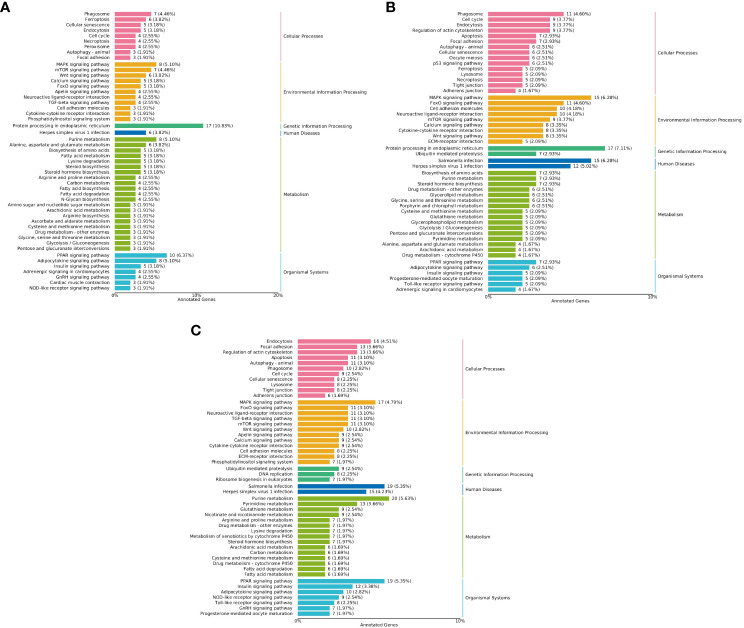
KEGG classification map of differentially expressed genes. Letters in corner marks **(A–C)** represent LS vs HS **(A)**, HS vs HL1 **(B)**, LS vs HL1 **(C)**, respectively. The left vertical coordinate is the name of the KEGG metabolic pathway, the right vertical coordinate represents the first-class classification name corresponding to the annotated pathway, and the horizontal coordinate is the number of genes annotated to the pathway and their proportion to the total number of genes annotated.

The results of KEGG enrichment analysis showed that, compared with the LS group on the whole fish meal diet, HSBMD induced changes in metabolic pathways. Insulin signaling pathway, adipocytokine signaling pathway, carbon metabolism, starch and sucrose metabolism, arginine and proline metabolism, glycolysis/gluconeogenesis and other pathways related DEGs were down-regulated. Terpenoid backbone biosynthesis, glutathione metabolism, steroid biosynthesis and protein processing in endoplasmic reticulum, PPAR signaling pathway and other pathways related DEGs were up-regulated. Including peroxisome proliferator-activated receptor alpha (PPARα), lectin, mannose-binding 2 (LMAN2) and other genes. In addition, immune-related pathways were also enriched with some DEGs, toll-like receptor signaling pathway, P53 signaling pathway, erbb signaling pathway, NOD-like receptor signaling pathway and Intestinal immune network for IgA production, and their DEGs were mostly down-regulated. In particular, MAPK signaling pathway and foxo signaling pathway related DEGs downregulation, including mitogen activated protein kinase kinase 6 (MKK6) and 5’-AMP-activated protein kinase, regulatory gamma subunit (PRKAG). The same HSBMD, LBP supplementation enriched DEGs mainly in pathways related to glucose and lipid synthesis and metabolism, including insulin signaling pathway, adipocytokine signaling pathway, glycolysis/gluconeogenesis, and glycolysis/gluconeogenesis. galactose metabolism, and their associated DEGs were upregulated. DEGs were up-regulated in P53 signaling pathway, erbb signaling pathway, nod-like receptor signaling pathway and other related pathways, and showed opposite results to LS vs HS. In addition, the MAPK signaling pathway and Foxo signaling pathway also showed opposite results. DEGs were mainly upregulated, and mitogen activated protein kinase 8 (MAP3K8) and forkhead box protein O1 (FOXO1) were also upregulated. The expression of toll-like receptor 3 (TLR3) and toll-like receptor 5 (TLR5) genes were down-regulated in toll-like receptor signaling pathway. Surprisingly, PPARα did not change significantly in the PPAR signaling pathway. However, its downstream lipid metabolism-related genes fatty acid-binding protein 1 (FABP1), lipoprotein lipase (LPL), long-chain acyl-coA synthetase (ACSL), carnitine o-palmitoyltransferase 1 (CPT-1) and phosphoenolpyruvate carboxykinase (PEPCK) were upregulated. On the other hand, in the LS vs HL1 group, the above pathways related to glucose and lipid synthesis and metabolism also changed accordingly, and the related DEGs were both up-regulated and down-regulated. In addition, TLR3 and TLR5 were also down-regulated in toll-like receptor signaling pathway, and mitogen activated protein-related genes were down-regulated in MAPK signaling pathway, but the downstream genes of the PPAR signaling pathway related to lipid metabolism were significantly up-regulated (**Figure 9**).

#### 3.3.4 RT-qPCR verification

**Figure 10:**
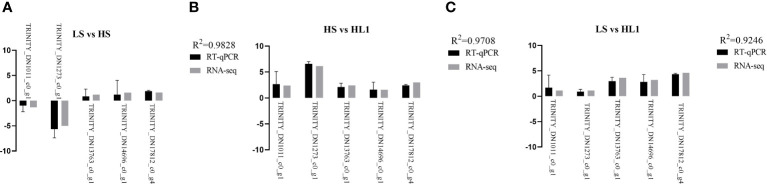
Relative expression levels (log2 fold change). Letters in corner marks **(A–C)** represent LS vs HS **(A)**, HS vs HL1 **(B)**, LS vs HL1 **(C)**, respectively.

After RNA-seq transcriptomic analysis of the data, in order to verify the accuracy of the transcriptomic results, RT-qPCR (biological repeat n=6) was used for verification analysis, that is, 5 common DEGs were randomly selected and pair-to-pair comparison was conducted among the three groups. The results confirmed that the five DEGs expression differences between the three groups were consistent with the RNA-Seq trend, where LS vs HS, R^2^ = 0.9828; HS vs HL1, R^2^ = 0.9708; LS vs HL1, R^2^ = 0.9246. The results further confirmed the reliability and authenticity of the results of RNA-Seq transcriptome analysis (**Figure 10**).

## 4 Discussion

HSBMD can cause a range of uncomfortable reactions in farmed animals. Yoshinaga et al. reported that fish with a large dietary intake of soybean meal showed growth retardation and physiological abnormalities, and cholesterol metabolism was also affected (37). Matulić et al. reported that the replacement of fish meal with soybean meal caused hepatic tissue degeneration in brown bullhead (*Ameiurus nebulosus L*) (38). In the results of this experiment, the addition of high soybean meal decreased the immune capacity of the spotted sea bass to a certain extent. And immune-related pathways such as toll-like receptor signaling pathway, P53 signaling pathway and erbB signaling pathway are also changed. In addition, compared with LS group, the hepatic morphology of HS group showed more fat vacuoles and balloon deformation. It was accompanied by severe hepatic cell injury, and the related pathways of glucose and lipid metabolism were also disturbed. These results suggest that HSBMD can cause serious tissue damage in the liver of spotted sea bass, which may be related to the decreased immunity and the glucolipid metabolic pathway. As mentioned in the introduction, this study focused on exploring whether LBP can mitigate these adverse effects or protect liver health. Therefore, this study will be discussed and focus on the two aspects of LBP to improve immunity and regulate glucose and lipid metabolism.

LZM is a natural antibacterial enzyme, which has lytic activity against both Gram-positive and Gram-negative bacteria, and can activate the complement system and phagocytes. Its activity is an important indicator of innate immunity in fish (39). Immunoglobulins (Ig) are glycoproteins that play a fundamental role in the adaptive immune system by recognizing antigens, and there are three types in teleost, namely IgM, IgD, and IgT/IgZ (40, 41). Among them, IgM is the main systemic antibody, involved in systemic immunity and mucosal immunity (42). ACP and AKP can change the surface structure of pathogens by hydrolysis, improve the body’s recognition and phagocytosis ability, and thus enhance disease resistance (43, 44). According to the results of this study, the addition of LBP significantly increased the activities of serum LYZ and IgM, hepatic ACP and AKP. Obviously, LBP improved the immunity of the spotted sea bass. Similarly, there are many studies that have reported the effect of LBP on improving immunity. Zhang et al. reported that LBP could improve the non-specific immunity of nile tilapia (*Oreochromis niloticus*) and reduce cell apoptosis (45). Zhu et al. reported that LBP induces dendritic cells to mature by stimulating the NF-κB signaling pathway mediated by Toll like receptor 2 (TLR2) and Toll like receptor 4(TLR4), so as to improve the immune capacity of the body (46). The research results of Zhang et al. show that LBP can significantly enhance the function of B cells and macrophages (47). It can be seen that LBP can enhance the body’s immunity through a variety of ways. In this experiment, TLR3 and TLR5 in AMPK signaling pathway were significantly down-regulated in the LBP addition group. TLR5 can combine with bacterial flagellin and cause obvious immune responses. The authors hypothesized that flagellin stimulation of membrane TLR5 leads to the induction of soluble TLR5S in the liver, which effectively binds to flagellin and then translocates it to membrane TLR5 factors in order to amplify danger signals in a positive feedback loop (48). In addition, Tsukada et al. found that soluble TLR5S was up-regulated 8 hours after attack by Vibrio or its purified flagellin (49). Therefore, we speculate that the down-regulation of TLR5 in this study is the result of reduced stimulation of the organism by bacteria or their flagellin. TLR3 is responsible for virus detection. Some studies have found the TLR3 gene has been over transcribed in rainbow trout after infection hematopoietic necrosis virus (*Oncorhynchus mykiss*) (50), and in zebrafish (*Danio rerio*) after infection with Snakehead Rhabdovirus (51), as well as in the Chinese rare minnow after challenge with the infectious Grass carp reovirus (*Gobiocypris rarus*) (52). Thus, similarly to TLR5, the down-regulation of TLR3 in this study may also be the result of reduced virus attack. On the other hand, NOD-like receptor signaling pathway (NLRS) also changed accordingly, and major DEGs expressions were up-regulated. NLRS plays an important role in innate immunity, and the up-regulation of its key DEGs means that LBP can enhance the immune capacity of the body through NLRS (53). In addition, apoptosis is an important mechanism for maintaining liver development and immune stability (54). The MAPK signaling pathway, foxo signaling pathway and P53 signaling pathway are all important pathways related to apoptosis. These pathways were altered by the addition of LBP, and these changes may be due to apoptosis to balance the liver damage caused by HSBMD. Based on the above changes of these pathways in different groups and the changes of their related DEGs, we speculate that the addition of LBP may activate or promote the apoptosis process. It has been reported that low-temperature induced inflammation and apoptosis may be the adaptive mechanism of antioxidant and immunity of freshwater drum (*Aplodinotus grunniens*) in freshwater drumfish (55). This suggests that there is a certain relationship between immunity and apoptosis, and this relationship may be regulated and balanced by the body itself. There is no doubt that this connection is complex and variable, but it is also of great study value. In addition, in this experiment, only when the supplemental level of LBP was 1 g/kg, the immunity of spotted sea bass was significantly improved, and the effects were also decreased when the supplemental level was increased. This suggests that excessive LBP supplementation may have negative effects or there may be immune fatigue to LBP. This is also worthy of further study in the future.

AST is mostly distributed in myocardium, liver and other tissues, while ALT is mainly present in liver cells. Under normal circumstances, only a small amount of AST and ALT are released into the blood, so the activity of AST and ALT in serum are very small. However, when hepatocytes are destroyed, the activity of AST and ALT in serum increases rapidly. Therefore, AST and ALT are used as relevant indexes to evaluate hepatic injury. The significant increase of serum AST and ALT activities induced by HSBMD in this study reflects that the liver has suffered serious damage. The results of hepatic morphology observation showed that HSBMD caused the increase of fat vacuoles and balloon deformation in the liver, and the liver cells were seriously damaged, which further supported the results of enzyme activity. In addition, transcriptomic sequencing revealed significant changes in pathways associated with glycolipid metabolism, including steroid biosynthesis, glycolysis/gluconeogenesis, glutathione metabolism and insulin signaling pathway. These may be the result of HSBMD. In particular, genes related to cholesterol biosynthesis were upregulated, suggesting that HSBMD may contribute to cholesterol deficiency. Fish meal contains more cholesterol than most plant-based protein ingredients (56). Therefore, HSBMD may have a lower cholesterol content, which may lead to a deficiency of cholesterol in the fish. This study reasonably hypothesized that the up-regulation of genes related to cholesterol synthesis was the result of poor adaptation to cholesterol. The same result was also found in the study of Yoshinaga et al. (37), who reported the results of transcriptomic analysis of the red seabream (*Pagrus major*) hepatopancreas, which ingests a large amount of soybean meal. Among them, Insulin signaling pathway, Carbon metabolism related DEGs down-regulation, Steroid biosynthesis, Glutathione metabolism related DEGs up-regulation. Interestingly, upregulation of the PPARα, which regulates lipid metabolism, was observed in this study. It has been demonstrated that gluconeogenesis is a direct target of PPARα and that PPARα has a significant impact on glucose homeostasis (57–59). Therefore, the up-regulation of PPARα may be an adaptive regulation of the body to the down-regulation of gluconeogenesis, but also a mechanism of the body’s self-regulation of glucose and lipid balance. This also indicates that the body may have produced a disorder in the production and metabolism of sugars and lipids, and this was also demonstrated by the changes in the glycolipid-related pathways in this experiment.

This study found that LBP can regulate the expression of downstream lipid metabolis-related genes in the PPAR signaling pathway, including FABP1, LPL, ACSL, CPT-1 and PEPCK, and they are all up-regulated. And after dietary intake of LBP, hepatic morphology was also improved to some extent, and serum ALT and AST activities were decreased. These results indicate that LBP has a positive effect on the regulation of lipid metabolism. Polysaccharides regulate lipid metabolism in many ways. One of the ways is that polysaccharides inhibit the body’s absorption of exogenous lipids and further affect the lipid metabolism process by binding with lipid molecules in the gastrointestinal tract or bile salts (60, 61). In general, the higher the molecular weight of the polysaccharide, the greater the hydrophobicity or viscosity, the stronger its binding ability (60). But interestingly, the study found that the lower the molecular weight of the polysaccharide, the stronger the binding ability of the bile acid (62). This indicates that the molecular weight of polysaccharide is not the only determinant of the binding ability of polysaccharide, and the composition and structure of polysaccharide may also be an important determinant. In addition, polysaccharide regulation can also regulate animal lipid metabolism by affecting intestinal microorganisms, regulating gene expression and related enzyme activity (61). The structure of intestinal flora plays an important role in the absorption and metabolism of lipids. Martinez-Guryn et al. showed that polysaccharides can regulate lipid metabolism by regulating intestinal flora (63). Furthermore, the animal intestinal microbiota and its metabolites also influence the expression of host-related genes. Zhang et al. reported that polysaccharide can activate intestinal HIF1α gene, and increase of this gene can not only promote the expression of local antimicrobial peptides, but also affect the expression of genes related to liver lipid metabolism through the gut-liver axis (64). Therefore, the molecular weight and composition of polysaccharide and the utilization of polysaccharide by fish can affect lipid metabolism. The structure and characterization of LBP are the prerequisite to reveal its function. At present, the primary structure of LBP includes molecular weight, the position of glycosidic bond, the type and proportion of monosaccharides, etc. These factors affect their biological activity to varying degrees (65). LBP mainly consists of five main structures: arabinogalactan, pectin polysaccharide, xylan, glucan, and other heteropolysaccharides (16, 66–68). These structures are mainly degraded by intestinal microorganisms to produce small molecule metabolites. These small molecule metabolites play a crucial role in host-gut microbiota interactions that help regulate gut health, lipid metabolism, and systemic immunity. For example, short-chain fatty acids are the main end metabolites produced during LBP fermentation and can regulate host physiology through multiple pathways (69–71). In addition, the intermediate products produced after the degradation of LBP by microorganisms may also be beneficial to the host. For example, oligosaccharides produced by microbial degradation of polysaccharides may have the ability to cross the vascular barrier and enter the systemic circulation to play an effect. It has been demonstrated that some oligosaccharides including 6’-sialyllactose, lacto-N-neotetraose and human milk 2’ -fucosyllactose can be absorbed into the plasma and thus reach the systemic circulation (72, 73). The monosaccharide composition, degree of polymerization and glycosidic linkage of LBP largely determine the regulatory effect of LBP on the host gut microbiota (74). Therefore, the structure and characterization of LBP, host gut microbiota, and metabolites have complex regulatory networks that need to be investigated through further *in vivo* and *in vitro* experiments.

On the other hand, this study found that GO and KEGG enrichment analysis and metabolism-related pathways were particularly active after ingestion of LBP. In particular, DEGs related to insulin signaling pathway, glycolysis/gluconeogenesis and galactose metabolism were upregulated. The monosaccharide composition of LBP used in this study includes Galactose, so when fish digest, absorb and utilize LBP, glycolysis/gluconeogenesis and galactose metabolism pathways naturally change. But using LBP directly to have this effect is minimal. First, the addition of LBP in this trial was small enough not to directly have this effect. Secondly, the ability of fish to directly absorb polysaccharides is limited, and the way to maximize the utilization is with the help of intestinal microorganisms and their metabolites. Therefore, this change may not only be caused by the absorption and utilization of LBP itself, but also may include the multi-pathway regulation of the body by LBP. Cai et al. reported that LBP decreased glucose uptake by down-regulating SGLT1 in Caco2 cells (75). Zhu et al. reported that LBP promoted the proliferation of pancreatic B cells, accelerated glucose metabolism and insulin secretion (76). Similarly, there are many studies on the hypoglycemic effect of LBP (77, 78). However, in this study, changes in insulin signaling pathway and other pathways related to sugar synthesis and metabolism were only found in the rich concentration of KEGG pathway, and changes in blood glucose were not detected. The subsequent LBP regulation mechanism of sugar metabolism in aquatic animals is worthy of further study. There is no doubt that sugar metabolism and lipid metabolism are related, or can regulate each other. This is undoubtedly a huge regulatory network, and the genes and pathways involved are also very complex. At present, the discussion on the mechanism of their interaction is still few, which needs to be further studied.

In summary, LBP has great potential in improving the immunity of aquatic animals, regulating glucose and lipid metabolism, and thus protecting hepatic health, and it is recommended to be widely used in aquaculture. However, as mentioned before, there is still a lack of reports on the negative effects of polysaccharides on farmed animals. These potential negative effects not only determine the optimum addition of polysaccharides, but also restrict the popularization and application of polysaccharides. In addition, combining polysaccharides with some anti-diabetic chemicals may have unexpected positive effects. However, it is necessary to consider whether the chemical drug shares a common target with the polysaccharide, resulting in competitive inhibition. Nevertheless, it is still a direction worth taking further. It is worth noting that the regulation of polysaccharide corresponding to lipid metabolism also has great potential in the treatment of fatty liver. And it is also worth paying attention to trying to study whether other herbal plant polysaccharides have the same excellent effect.

## 5 Conclusion

In conclusion, the addition of LBP in the diet can improve the immune capacity of spotted sea bass and affect the immune and glycolipid metabolism related pathways, so as to improve the damage of liver and the imbalance of glycolipid metabolism caused by high soybean meal in the diet to a certain extent, and thus protect the liver health of spotted sea bass. This result reveals the potential of LBP to enhance immunity and improve glucose and lipid metabolism of aquatic animals, and subsequent studies can focus on the negative effects of LBP and development of related targets.

## Data availability statement

The datasets presented in this study can be found in online repositories. The names of the repository/repositories and accession number(s) can be found below: NCBI SRA, PRJNA1070061.

## Ethics statement

The animal study was approved by Jimei University Animal Ethics Committee. The study was conducted in accordance with the local legislation and institutional requirements.

## Author contributions

LL: Writing – original draft, Writing – review & editing. YZ: Writing – review & editing. ZH: Writing – review & editing. ZYL: Writing – review & editing. HQ: Writing – review & editing. HL: Writing – review & editing. SZ: Writing – review & editing. LK: Writing – review & editing. JM: Writing – review & editing. ZBL: Data curation, Funding acquisition, Methodology, Resources, Writing – review & editing.
